# Idiopathic Intracranial Hypertension in Neonates, Infants, and Toddlers

**DOI:** 10.3390/jcm14145084

**Published:** 2025-07-17

**Authors:** Efstathios Beys-Kamnarokos, Ioannis Mavridis

**Affiliations:** 1 Pediatric Neurosurgery Unit, Department of Neurosurgery, School of Medicine, Democritus University of Thrace, University General Hospital of Alexandroupolis, Dragana Campus, 68100 Alexandroupolis, Greece; efstathios.beys-kamnarokos@evkb.de; 2 Department of Neurosurgery, Protestant Hospital of the Bethel Foundation, Medical School and University Medical Center OWL, Bielefeld University, Campus Bielefeld-Bethel, 33617 Bielefeld, Germany

**Keywords:** idiopathic intracranial hypertension (IIH), papilledema, pseudotumor cerebri syndrome, neonates, infants, toddlers, diagnostic criteria

## Abstract

Idiopathic intracranial hypertension (IIH) with (IIHWP) and without papilledema (IIHWOP) is characterized by increased cerebrospinal fluid (CSF) pressure and no evident cause, mostly affecting obese women of childbearing age and possibly leading to vision loss. However, in neonates, infants, and toddlers, these conditions remain understudied entities. This review investigates clinical features, risk factors, treatments, and outcomes to inform their care. From 2278 publications found in PubMed, 2974 in Scopus, and 1684 in the Web of Science Core Collection, 104 relevant articles were analyzed. Among 300 cases, 48.3% were male and 26.0% female, with 43.0% meeting the modified Dandy criteria. Typical signs and symptoms, besides papilledema (23.0%) or its absence (49.0%), included bulging fontanelle (67.7%), irritability (34.3%), vomiting (33.0%), and fever (18.3%). The most triggering factors were medications (35.3%) and infections (15.0%). The mean CSF opening pressure was 35.1 cm H_2_O, ranging from 9.5 to 77 cm H_2_O. Main treatment options were lumbar punctures (72.7%), discontinuation of triggering medications (26.3%), acetazolamide (18.7%), and corticosteroids (7.7%); 3.0% required shunting. Unlike in adults, males were more commonly affected, and papilledema was less frequent. Most cases resolved with conservative treatment. A nosological distinction between IIHWP and IIHWOP seems unlikely. Considering our findings and age-specific CSF pressure limits, new diagnostic criteria are proposed.

## 1. Introduction

Idiopathic intracranial hypertension (IIH), first described by Heinrich Quincke in 1893 [1] as meningitis serosa and named pseudotumor cerebri syndrome in 1904 by Max Nonne [2], also known as benign or primary intracranial hypertension, is mainly characterized by increased cerebrospinal fluid (CSF) pressure and no evident cause [3]. It mostly affects obese women of childbearing age and is usually manifested with headache, nausea, auditory problems, transient visual disturbances, papilledema, and permanent visual loss [4,5], although up to 25% of patients are asymptomatic [6,7].

The worldwide incidence of IIH in the general population ranges from 0.03 to 7.8 per 100,000 people per year [4,5]. In obese women of childbearing age, it raises up to 12–20 per 100,000 people per year [8]. The prevalence has been estimated in only one publication, reporting 76 per 100,000 people in a local population [4]. The incidence of pediatric IIH is slightly lower, with 0.63–0.9 per 100,000 children (age < 18 years) per year [9,10,11]. For neonates (0–1 month), infants (1–12 months), and toddlers (12–36 months), no separate data are available.

Idiopathic intracranial hypertension without papilledema (IIHWOP) constitutes a rare variant. There is one cohort study describing 5% of IIH cases lacking papilledema [12]. Moreover, two studies investigating chronic daily headache in IIH found an absence of papilledema in 10% and 14% of cases, respectively [13,14]. Although IIHWOP is phenotypically very similar to IIH with papilledema (IIHWP), it is still unclear whether they are nosologically distinct [15].

Most studies and reviews to date have focused on adults, leading to the development of diagnostic criteria and treatment guidelines tailored to that population [16,17,18,19]. Pediatric IIH and IIHWOP, on the other hand, represent under-studied entities. Especially in neonates, infants, and toddlers, only isolated case reports or small case series have been described in the literature. This review focuses on this age group and aims to examine the literature for clinical presentation, risk factors, treatment, and outcomes in order to provide new data applicable to and potentially guiding clinical practice.

## 2. Materials and Methods

We systematically searched the PubMed (U.S. National Library of Medicine, Bethesda, MD, USA), Scopus (Elsevier, Philadelphia, PA, USA), and the Web of Science Core Collection (Clarivate, Philadelphia, PA, USA) databases for IIH cases < 36 months old, using the following search strings.

### 2.1. For PubMed and Web of Science Core Collection

Idiopathic intracranial hypertension (child* OR pediatric OR infant* OR toddler) OR benign intracranial hypertension (child* OR pediatric OR infant* OR toddler) OR primary intracranial hypertension (child* OR pediatric OR infant* OR toddler) OR pseudotumor cerebri (child* OR pediatric OR infant* OR toddler).

### 2.2. For Scopus

(TITLE-ABS-KEY (idiopathic AND intracranial AND hypertension AND (child* OR pediatric OR infant* OR toddler))) OR (TITLE-ABS-KEY (benign AND intracranial AND hypertension AND (child* OR pediatric OR infant* OR toddler))) OR (TITLE-ABS-KEY (primary AND intracranial AND hypertension AND (child* OR pediatric OR infant* OR toddler))) OR (TITLE-ABS-KEY (pseudotumor AND cerebri AND (child* OR pediatric OR infant* OR toddler))).

**Figure 1:**
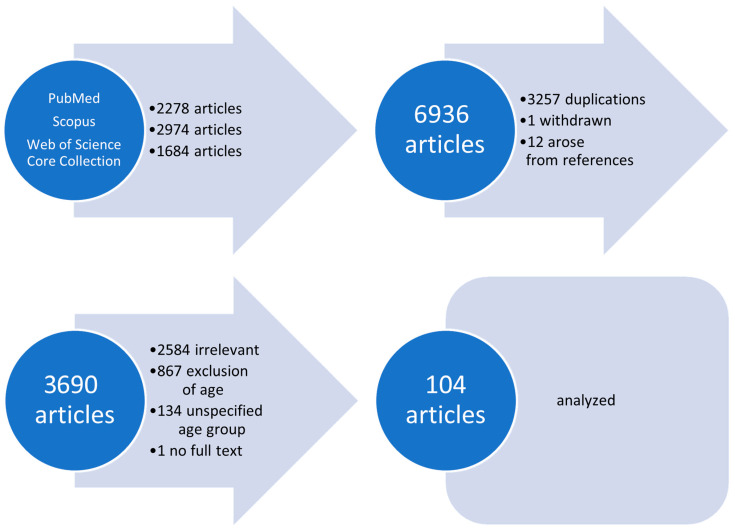
Flow chart of the literature review. A total of 104 publications were finally included in the analysis.

PubMed search resulted in 2278 entries, Scopus in 2974, and the Web of Science Core Collection in 1684. All articles published before April 2025 were screened. One publication was withdrawn, and 3257 articles were listed in more than one database. Titles and abstracts were examined and full texts of articles regarding the targeted age group, 0 to 36 months, were studied. Moreover, references of relevant articles were considered, from which 12 more publications arose. As a result, 3690 articles were investigated. A total of 867 papers were excluded due to the patients’ age, and 134 articles due to commingling of the targeted age group with older patients without separating their data. In addition, 2584 articles were irrelevant, and for one relevant article, no full text could be found. As a result, 104 publications describing IIH cases were included in the analysis (Figure 1). No restrictions regarding language or date of publication were set, nor were diagnostic criteria for exclusion applied.

**Figure 2:**
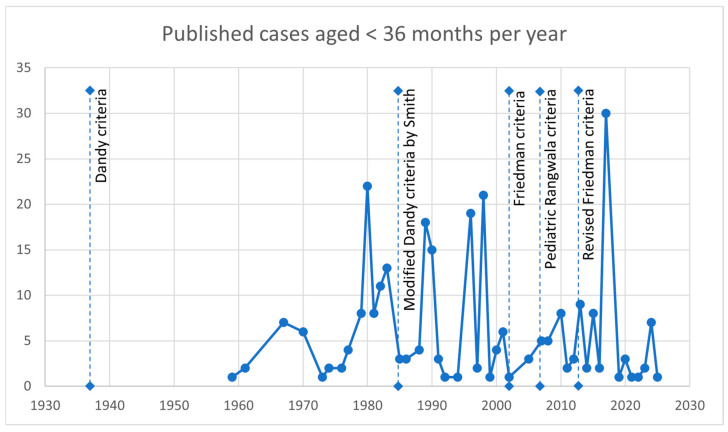
Timeline with the number of published cases aged < 36 months per year and the time points of establishing diagnostic criteria.

Since no restrictions regarding the date of publication were made, and consequently many articles published before the establishment of diagnostic criteria were included (Figure 2), in the first instance none of the five definitions were used: the Dandy criteria from 1937 [20], the modified Dandy criteria by Smith from 1985 [16], the Friedman criteria from 2002 [17], the pediatric Rangwala criteria from 2007 [18], and the revised Friedman criteria from 2013 [19]. Afterwards, the modified Dandy criteria were retrospectively applied as the first well-established criteria with the probable lowest inclusion threshold.

## 3. Results

**Figure 3:**
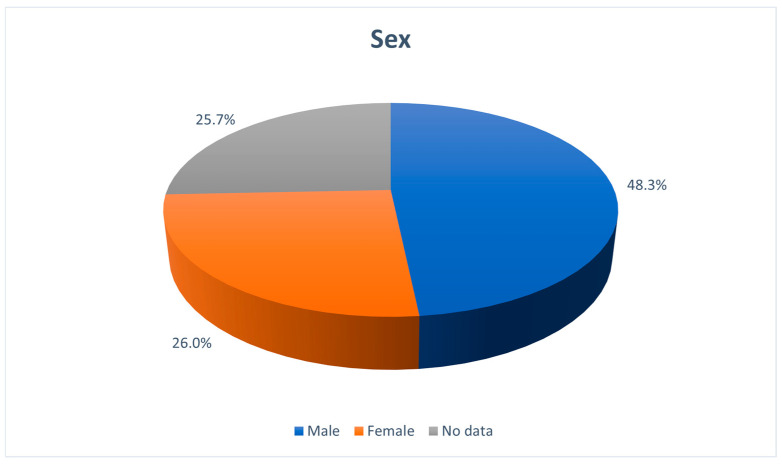
Sex in percentages of patients.

As presented in the supplementary table (Supplementary Materials: Table S1), 104 articles describe 300 neonates, infants, and toddlers diagnosed by the authors with IIHWP or IIHWOP [3,6,21,22,23,24,25,26,27,28,29,30,31,32,33,34,35,36,37,38,39,40,41,42,43,44,45,46,47,48,49,50,51,52,53,54,55,56,57,58,59,60,61,62,63,64,65,66,67,68,69,70,71,72,73,74,75,76,77,78,79,80,81,82,83,84,85,86,87,88,89,90,91,92,93,94,95,96,97,98,99,100,101,102,103,104,105,106,107,108,109,110,111,112,113,114,115,116,117,118,119,120,121]. The youngest patient was 15 days old [77]. A total of 129 (43.0%) of them met the modified Dandy criteria. The majority, 48.3%, were male, and 26.0% female (Figure 3). In the rest of the cases (25.7%), no sex-related data were available. Males were affected more often than females (1.85:1).

**Figure 4:**
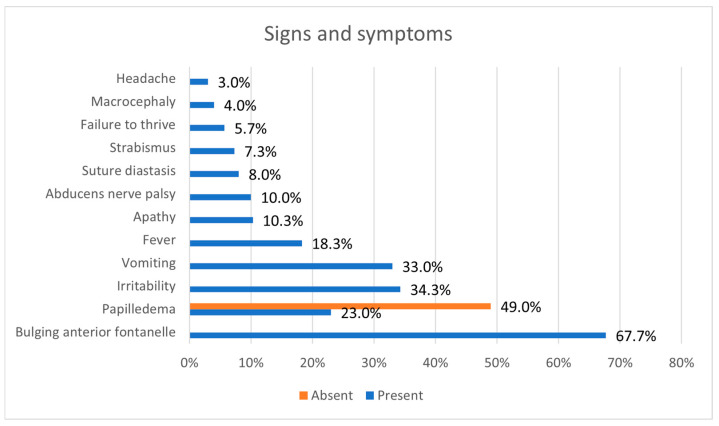
Signs and symptoms in percentages of patients.

Papilledema was described in 23.0% of cases and was absent in 49.0% of cases. In the rest of the cases, no data were available. The signs and symptoms included bulging anterior fontanelle in 67.7%, irritability in 34.3%, vomiting in 33.0%, fever in 18.3%, apathy in 10.3%, abducens nerve palsy in 10%, suture diastasis in 8%, strabismus in 7.3%, failure to thrive in 5.7%, macrocephaly in 4%, and headache in 3% (Figure 4). Except for one patient aged 14 months [39], patients with a bulging fontanelle were infants. The average number of signs and symptoms was 2.95.

**Figure 5:**
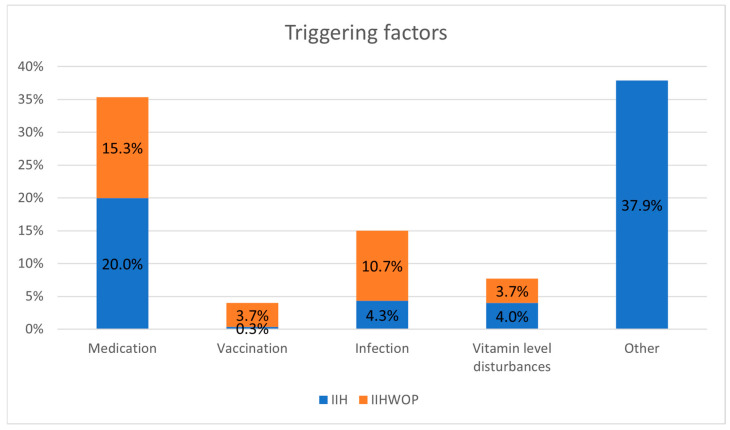
Triggering factors in percentages of patients.

In total, 15.0% of IIH cases were associated with infection, mostly relating to the upper respiratory tract or otitis media, and rarely to roseola infantum [29,50,54,73,90], with 71.1% of them classified as IIHWOP. A total of 35.3% were related to medication, with 43.4% of them as classified as IIHWOP, and 35.8% of them due to nalidixic acid. Other medications were steroids [25,34,43,44,82,86,97,98,105], antibiotics [30,31,34,60,66,71,72,85,95,104], β2 agonists [30], and human growth hormone [108]. Additionally, 7.7% of cases were triggered by vitamin level disturbances, with 47.8% of them classified as IIHWOP. A total of 4% were associated with vaccination, with 47.8% of them classified as IIHWOP. Other conditions, such as anemia [87,102,113,117], cystic fibrosis [30,102], hypophosphatasia [21,47,79,117,119], elevated systemic venous pressure [100], soybean formular substitution [73], and retinoblastoma [81], were also occasionally mentioned as possible triggering factors. In one case, IIH appeared after a filum lysis for tethered cord syndrome, with the authors suspecting disruption in CSF flow dynamics, although an infection-related appearance could not be excluded [55]. Another case was described following head injury, and another case yet to be correlated with maple syrup urine disease [67,77] (Figure 5).

**Figure 6:**
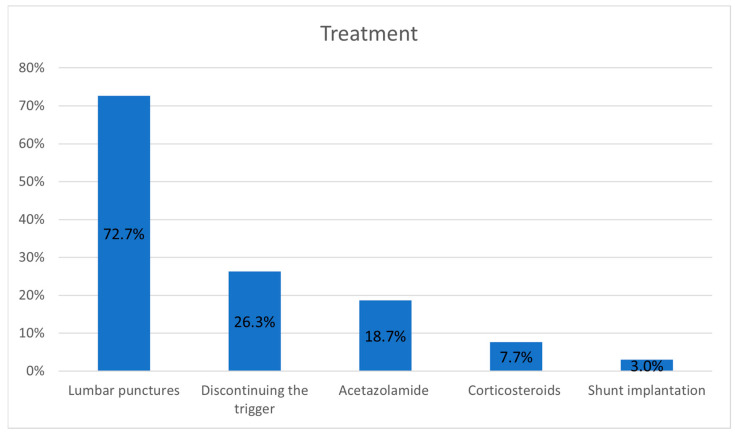
Main treatment options in percentages of patients.

Mean CSF opening pressure was 35.1 cm H_2_O, with a range of 9.5–77 cm H_2_O. Separated into IIHWP and IIHWOP, an average of 37.0 cm H_2_O vs. 31.4 cm H_2_O was calculated. The main treatment options were lumbar punctures in 72.7% of cases, discontinuation of the triggering medication in 26.3%, acetazolamide in 18.7%, corticosteroids in 7.7%, and treatment of IIH-related infection. Only 3.0% of cases needed some kind of shunt implantation (Figure 6). In patients fulfilling the modified Dandy criteria, acetazolamide was used in 42.2% and corticosteroids in 11.7% of cases. Remarkably, in some cases IIH was self-limiting without treatment. The time to symptom relief after hospitalization ranged from 24 h to several days. The patients were free of symptoms usually several days later, although some cases lasted for months or, extremely rarely, for over a year.

The average CSF pressure in IIHWP patients with a bulging fontanelle was 38.2 cm H_2_O, and in IIHWP patients without a bulging fontanelle it was 36.9 cm H_2_O. The average CSF pressure in IIHWOP patients with a bulging fontanelle was 31.3 cm H_2_O, and in IIHWOP patients without a bulging fontanelle it was 31.6 cm H_2_O. Although age the associated possible suture closure were not considered in this observation.

Ιn pre-pubertal children, no correlation between CSF pressure and severity of clinical symptoms has been found [49]. In our study and focused age group, a slight correlation was noted between CSF pressure and the quantity of clinical symptoms in IIHWP and IIHWOP. Also, a worsening of symptoms at nighttime and/or in the horizontal position with subsequently increased intracranial pressure (ICP) has been described [100].

## 4. Discussion

Uncertainty surrounds the fundamental comprehension of the underlying pathophysiology of IIH. Disruptions in the veno-glymphatic, neuroendocrine, and hormonal systems as well as inflammatory processes are believed to affect in one way or another the CSF system in the form of secretion or absorption disturbances, leading to raised ICP [122]. All of our recorded possible triggering factors could fit into this concept and support every of these hypotheses.

As already mentioned, 43.0% of cases met (retrospectively) the modified Dandy criteria. In the remaining cases, either a lumbar puncture or an imaging study was omitted, mostly because the symptoms resolved rapidly with the initial therapeutic medication or discontinuation of the triggering factor, and a further diagnostic test to confirm the diagnosis was considered obsolete, keeping in mind the difficulty and effort needed for lumbar puncturing or obtaining magnetic resonance images in neonates, infants, and toddlers.

Another conspicuity in the studied age group is that males are, in contrast to adults, more often affected than females [8], a finding that is difficult to interpret. Since androgen excess certainly seems to play a role in IIH, as observations in a cohort [123], transgender patients [124,125], and rat models [126,127] showed, we speculate that the increasing testosterone levels during minipuberty (transient activation of the hypothalamic–pituitary–gonadal axis during the first 6 months of life) in boys [128] could be at least partially responsible for that difference. At this point, we would like to note that, in our analysis, the sex was known in 223 (48.3% + 26.0% = 74.3%) out of 300 patients, and this part was taken into consideration. A severely different sex distribution in the unknown remaining 25.7% that would significantly change the overall sex distribution is statistically unlikely and hypothetical and therefore a conclusion is justified.

Regarding fever, it is noteworthy that 18.3% of IIH cases were febrile while only 15.0% were triggered by an infection. So, at least in 3.2%, a systemic response of the organism to elevated ICP could be assumed.

In the literature, the closure of the anterior fontanelle has been observed between 4 and 26 months of age, mainly between 10 and 20 months. Delayed closure may reflect a wide variety of possible underlying pathologies, including elevated ICP among others [129]. This would be consistent with the fact that 17 of 24 (70.8%) for IIH, and 10 of 14 (71.4%) for IIHWOP, in our study’s registered cases with suture diastasis were noted with a bulging anterior fontanelle as a sign of raised ICP, probably protracting the closure process. On the contrary, premature craniosynostosis, which leads to elevated ICP due to decreased intracranial volume and thus mimics symptoms of IIH, should be excluded [130,131]. The same applies to causes of elevated intracranial venous pressure, which results in intracranial hypertension due to impaired CSF absorption [132].

Papilledema is pathophysiologically a result of axoplasmic stasis within retinal ganglion cell axons. Increased ICP compresses the short posterior ciliary arteries and the retrolaminar portion of the optic nerve within the optic nerve sheath, producing intraaxonal edema [133]. If open fontanelles are present, higher pressures transmit first to them rather than to the optic nerves [24]. Furthermore, an open anterior fontanelle can compensate to some extent for elevated ICP, since more space and volume can accrue, leading to a bulging fontanelle. So, it does not seem surprising that the proportion of IIHWOP cases at this age group is in at least 49.0%, and, respectively, 45.7% in the group fulfilling the modified Dandy criteria, higher than in older children and adults [12,13,14]. Additionally, the organism could become more accustomed to a slow increase in CSF pressure, thus avoiding papilledema and/or a bulging fontanelle. In consequence, we do not share the opinion of two nosologically distinct entities regarding IIHWP and IIHWOP.

Aside from that, the normal pediatric ICP range has not been clarified entirely yet, especially for neonates, infants, and toddlers. Recently, 5 cm H_2_O has been mentioned as the upper normal limit for infants with hydrocephalus [134]. Rangwala specified 7.6 cm H_2_O for neonates in his criteria [18]. In Greenberg’s *Handbook of Neurosurgery*, 10th edition, 8.2 cm H_2_O for infants and 9.5 cm H_2_O for young children are mentioned [135]. These limits correlate with our findings, since the lowest registered elevated CSF pressure was 9.5 cm H_2_O and the second lowest 10 cm H_2_O, all of them fulfilling the modified Dandy criteria. In any case, the CSF pressure limits in the previously proposed criteria are too high for the targeted age group, thus risking underdiagnosis. The need for new pediatric criteria has also been formulated recently by other authors [100].

With all this in mind, we propose the following criteria for diagnosing pediatric IIH in neonates, infants, and toddlers (Table 1).

Further we agree with a prior publication that the diagnostic criteria for IIH should not be too strict [136], since the main aim is to identify patients at risk for future vision loss, especially at such a young age. A timely intervention in the early stages of illness with minor symptoms is all the more important.

This review is, to the best of our knowledge, the first one focused on neonates, infants, and toddlers with IIH to such an extent. It analyzed data regarding the clinical picture, risk factors, treatment, and outcomes, and it provided results useful in the clinical management of these patients.

## 5. Limitations

In this review, cases have been included mostly from older publications, where diagnosis of IIH had been set by their treating physician and did not necessarily meet any of the known criteria. The modified Dandy criteria were retrospectively applied by the authors of the review, often posing difficulties due to incomplete case descriptions. The sex, status of papilledema, or CSF pressure, for example, were in many cases not mentioned. The possibilities and quality of radiological imaging for excluding other pathologies varied. In the early years, only ventriculography and pneumoencephalography were available until ultrasonography, computer tomography, and later magnetic resonance imaging became available. All in all, it was difficult to summarize and compare data from many decades regarding a pathology which still has not been fully understood, with the latter justifying our purpose.

## 6. Conclusions

In contrast to adults, males were affected by IIH more often than females, and papilledema was significantly more rarely present. Discontinuation of the triggering factor, treatment with lumbar punctures, acetazolamide, and corticosteroids usually resolved the symptoms, so that a shunt implantation was only rarely necessary. The hypothesis of two nosologically distinct entities regarding IIHWP and IIHWOP seems unlikely. Moreover, the CSF pressure limits in the previously proposed criteria seem too high for the targeted age group, thus risking underdiagnosis. Therefore, considering our findings and the deviating CSF pressure limits for this age group, new diagnostic criteria for neonates, infants, and toddlers are proposed. Further research with prospective data is needed for a better understanding of IIH in early childhood. A timely intervention in the early stages of a potentially severe illness with occasionally minor symptoms, like IIH, is all the more important for the affected children.

## Figures and Tables

**Table 1:** Proposed diagnostic criteria for IIH in neonates, infants, and toddlers. CSF: cerebrospinal fluid, MRI: magnetic resonance imaging, MRV: magnetic resonance venography.

1. <36 Months Old
2. CSF pressure in: a. Neonates: >7.6 cm H_2_O b. Infants: >8.2 cm H_2_O c. Toddlers: >9.5 cm H_2_O
3. Normal age-appropriate CSF composition
4. If symptoms, reflecting intracranial hypertension
5. Normal neurological examination except cranial nerve abnormalities
6. At least one of: a. Papilledema b. Sixth nerve palsy c. Bulging fontanelle d. Directly prior infection or new medication intake and regressive symptoms within 24 h after discontinuation
7. No other identified cause of intracranial hypertension on MRI/MRV except for transverse venous sinus stenosis

## Data Availability

The original contributions presented in this study are included in the article/Supplementary Materials. Further inquiries can be directed to the corresponding author.

## Supplementary Materials

The following supporting information can be downloaded at: https://www.mdpi.com/article/10.3390/jcm14145084/s1, Table S1: Summary of 104 publications including 300 IIH cases aged up to 36 months. References [3,6,21,22,23,24,25,26,27,28,29,30,31,32,33,34,35,36,37,38,39,40,41,42,43,44,45,46,47,48,49,50,51,52,53,54,55,56,57,58,59,60,61,62,63,64,65,66,67,68,69,70,71,72,73,74,75,76,77,78,79,80,81,82,83,84,85,86,87,88,89,90,91,92,93,94,95,96,97,98,99,100,101,102,103,104,105,106,107,108,109,110,111,112,113,114,115,116,117,118,119,120,121] are cited in Supplementary Materials.

## Abbreviations

The following abbreviations are used in this manuscript:

CSF	Cerebrospinal fluid
ICP	Intracranial pressure
IIH	Idiopathic intracranial hypertension
IIHWOP	Idiopathic intracranial hypertension without papilledema
IIHWP	Idiopathic intracranial hypertension with papilledema
MRI	Magnetic resonance imaging
MRV	Magnetic resonance venography
